# Umbilical cord blood: A promising source for allogeneic CAR-T cells

**DOI:** 10.3389/fonc.2022.944248

**Published:** 2022-07-29

**Authors:** Dian-Dian Liu, Wei-Cong Hong, Kun-Yin Qiu, Xin-Yu Li, Yong Liu, Li-Wen Zhu, Wei-Xin Lai, Han- Chen, Hua-Qing Yang, Lu-Hong Xu, Jian-Pei Fang

**Affiliations:** ^1^ Department of Pediatrics, Sun Yat-sen Memorial Hospital, Sun Yat-sen University, Guangzhou, China; ^2^ Key Laboratory of Malignant Tumor Gene Regulation and Target Therapy of Guangdong Higher Education Institutes, Sun Yat-sen University, Guangzhou, China

**Keywords:** chimeric antigen receptor T cells, umbilical cord blood, peripheral blood, acute lymphoblastic leukemia, T-lymphocyte subsets, NCG-immunodeficient mice

## Abstract

Chimeric antigen receptor T (CAR-T) cell therapy is an effective treatment for relapsed and refractory acute lymphoblastic leukemia (R/R ALL). However, autologous CAR-T cells derived from patients with B-ALL often show poor amplification ability, exhaustion, and anergy. To overcome these limitations, allogeneic CAR-T cells may be used as effective substitutes; however, which source would be the best substitute is unclear. In this study, we compared the immunophenotype and antitumor efficacy of anti-CD19 CAR-T cells derived from healthy donor cord blood (CB), healthy donor peripheral blood (PB), and PB of B-ALL patients [PB (patient)] *in vitro* and NOD-Prkdcem26cd52Il2rgem26Cd22/Nju (NCG)-immunodeficient mice, respectively. The results revealed that CAR-T cells derived from healthy donor CB and PB showed a higher proportion of naive T cells and longer tumor suppression in tumor-bearing mice than those of PB (patient). PB (patient) CAR-T cells had a higher proportion of regulatory T cells (Treg cells) and released high levels of interluekin-10 (IL-10), which also suggest a poor prognosis. Thus, CAR-T cells derived from healthy donors have better antitumor efficacy than CAR-T cells derived from PB (patient), and CB may be a good source of allogeneic CAR-T cells.

## Introduction

The chimeric antigen receptor T (CAR-T) cell is a type of genetically modified T cell that links a single-chain antibody that can recognize tumor-specific antigens, a transmembrane domain, and an intracellular T-cell activation domain *in vitro* (1, 2). First-generation CAR-T cells only have the antibody single-chain variable fragment (scFv) and the T cell-activating domain, the zeta chain of the CD3 complex (CD3z), which shows poor antitumor efficacy in clinical trials due to limited expansion and persistence (3). Based on the first generation, the second and third generations of CAR-T cells solved this problem by adding costimulatory signal domains (CD28, 4-1BB, ICOS, and OX-40) to enhance their signaling capability and sustain a T-cell response to antigen exposure (4). Furthermore, the fourth generation of CAR-T cells could secrete immunomodulatory cytokines [IL-12/IL-15/granulocyte-macrophage colony stimulating factor (GM-CSF)] (5).

CAR-T cells demonstrated excellent antitumor properties in many clinical trials, especially in CD19^+^ acute lymphoblastic leukemia (ALL) (6–8). However, there are many unresolved issues regarding the use of CAR-T cells. Currently, autologous CAR-T cells derived from cancer patients are commonly used in clinical treatment. However, patients with relapsed and refractory (R/R) ALL or other hematological malignancies such as chronic lymphoblastic leukemia, multiple myeloma, and diffuse large B-cell lymphoma still undergo intensive lymphocytotoxic chemotherapy, rendering T cells “dysfunctional,” “anergic,” or difficult to collect (9, 10). Therefore, using healthier T cells to make off-the-shelf products could further enhance the efficacy of CAR-T therapy. Currently, universal CAR-T cells (UCAR-T cells) from healthy donors or allogeneic CAR-T cells combined with hematopoietic stem cell transplantation are in the spotlight and are expected to end the plight (11, 12).

Allogeneic CAR-T cells derived from peripheral blood (PB) T cells are most commonly used, but the collection process is harmful to the donor. Cord blood (CB) is a product of childbirth, does little damage to the donor, and contains an abundance of immune cells, making it a promising source for cellular immunotherapy (13, 14). However, only a small number of studies have reported on the antitumor efficacy of CAR-T cells derived from CB, and the differences in immunophenotype and function between CAR-T cells derived from CB and PB are not fully understood. Therefore, in this study, we compared the T-cell subsets and functional characterization of T cells derived from CB and PB of healthy donors, with PB of B-ALL patients [PB (patient)] to provide a scientific basis for better use of CAR-T cells derived from CB in the future.

## Materials and methods

### Patients and healthy donors included in the study

We obtained PB samples from 10 healthy donors and 15 B-ALL patients and (cryopreserved) CB samples from 22 healthy donors. All patients received no chemotherapy or immunosuppression treatment for at least 2 weeks prior to blood collection, had no serious active infection, and had no anti-CD25 monoclonal antibody treatment during collection. The most recently measured bone marrow minimal residual disease levels of ALL patients were <0.01%. This study was approved by the institutional review board of Sun Yat-sen Memorial Hospital, Clinical Research Center. Informed signed consent was obtained from all patients who participated in the study.

### Immunostaining and flow cytometry analysis of T-cell subsets

We analyzed the proportions of TN(CD45RA+CCR7+), TCM(CD45RA-CCR7+), TEM(CD45RA-CCR7-), TEMRA(CD45RA+CCR), and Treg (CD4+CD25+CD127-) in T cells and detected the expression of PD-1, LAG3, and CD69 in T cells by flow cytometry. T cells were stained with fluorescent-labeled antibodies against CD3 (Perp-Cy5.5-conjugated mouse anti-human antibody, clone UCHT1), CD4 (FITC-conjugated mouse anti-human antibody, clone 13B8.2), CD8 (BV510-conjugated mouse anti-human antibody, clone SK1), CD45RA (APC-CY7-conjugated mouse anti-human antibody, clone HI100), CCR7 (APC-conjugated mouse anti-human antibody, clone G043H7), CD69 (BV421-conjugated mouse anti-human antibody, clone FN50), CD25 (PE-conjugated mouse anti-human antibody, clone B1.49.9), PD-1 (PE-conjugated mouse anti-human antibody, clone EH12.2H7), LAG3 (BV421-conjugated mouse anti-human antibody, clone 747-530), and CD127 (BV421-conjugated mouse anti-human antibody, clone R34.34); all antibodies were purchased from BD Biosciences, Franklin Lakes, NJ, USA. Cytofifix/CytopermTM Plus kit (BD Biosciences) was used for cell membrane fixation and permeabilization. Flow cytometry analysis was performed using a FACS CANTO II flow cytometer. The fluorescence activated cell sorting (FACS) Diva software (BD Biosciences) or Kazula Analyzer Software (Beckman Coulter, Brea, CA, USA) was used to analyze the data.

### T-cell collection and generation of CD19 CAR-T cells

We constructed 1928zT2 CARs with a CAR linking FMC63-scFv, CD28 transmembrane, endodomain, CD3ζ signaling domain, and Toll/interleukin-1 receptor domain (amino acid 639–784) of TLR2 (1). Five samples from each source were selected for *in vitro* and *in vivo* experiments. Mononuclear cells were isolated from samples by centrifugation using a Ficoll gradient (Eurobio, Les Ulis, France, CMSMSL01-01). T cells were isolated and stimulated by MACS GMP TransAct CD3/CD28 Kit RUO and IL-2 (200 IU/ml) for 48 h and then transfected into third-generation CAR-T cells by lentivirus carrying 1928zT2 CARs. Transduced cells were then cultured in media containing Roswell Park Memorial Institute (RPMI) 1640 (Gibco-BRL, Gaithersburg, MD, USA), 10% fetal bovine serum (Hyclone Laboratories Inc., Logan, UT, USA), 1% l-glutamine (Invitrogen, Carlsbad, CA, USA), and IL-2 (200 IU/ml) for 9–11 days before subsequent analysis. The efficiency of transfection was evaluated by green fluorescent protein (GFP).

### Cell line generation

The Nalm6-GL and K562-GL cell lines were maintained in RPMI 1640 (Gibco-BRL) supplemented with 10% fetal bovine serum. 239T cells were maintained in Dulbecco’s Modified Eagle Medium (Gibco-BRL) and 10% fetal bovine serum.

### *In vitro* assays of CAR-T cell-mediated cytotoxicity and cytokine release

CAR-T cells were sorted by the FACS Aria cell sorting system (FACSCalibur, BD) using GFP. To assess CAR-T cell cytotoxicity, Nalm6-GL and K562-GL cell lines expressing luciferase were plated at 2 × 10^4^ cells/well in black 96-well plates (Corning Costar, Corning, NY, USA) and cultured with T cells/well in an effector to a target ratio of 1:1, 1:2, 1:4, 1:8, and 1:16. Before coculture, T cells were washed three times with phosphate-buffered saline. After 18 h of being cocultured with Nalm6-GL, the medium was aspirated for IL-2, IL-6, IL-10, tumor necrosis factor alpha (TNF-α), and interferon gamma (IFN-γ) secretion analysis using the BD™ Cytometric Bead Array (CBA) Human Th1/Th2 Cytokine Kit II (BD Biosciences). The viability of the cancer cells was determined by measuring luminescence with a GloMax Discover Plate Reader (Promega Madison, WI, USA) after adding luciferin (150 μg/ml) to every well.

### *In vivo* antitumor activity

In our study, female NOD-Prkdcem26cd52Il2rgem26Cd22/Nju (NCG) mice were purchased from the Model Animal Resource Information Platform of Nanjing University (Nanjing, P. R. China) and kept under pathogen-free conditions in the animal vivarium at the Animal Laboratory Center, Sun Yat-sen University. All animal experiments were performed according to the guidelines of the Committee on Animal Use and Care of Sun Yat-sen University.

Four- to eight-week-old NCG mice were infused with 5 × 10^5^ Nalm6-GL cells intravenously. Five days after tumor cell infusion, the tumor burden of mice was monitored using the Xenogen-IVIS Imaging System (Caliper Life Sciences, Hopkinton, MA, USA), and subsequently, the mice were divided into the following six groups, with five mice each, based on tumor burden: CB CAR-T, PB CAR-T, PB (patient) CAR-T, CB wild type (WT), PB WT, and PB (patient) WT. On day 0, in the CB CAR-T, PB CAR-T, and PB (patient) CAR-T groups, mice were intravenously injected with 1 × 10^7^ CAR-T cells. In the CB WT, PB WT, and PB (patient) WT groups, mice were intravenously injected with 1 × 10^7^ T cells. After infusion with T cells, the tumor signals of the mice were monitored using the Xenogen-IVIS Imaging System (Caliper Life Sciences) every 7 days until the end of the experiment. On days 14 and 28, the proportions of human T cells and Nalm6-GL cells in the PB of mice were measured using a flow cytometer. When the mice became paralyzed and were unable to eat, they were euthanized. At the end of the experiment, the surviving mice were sacrificed, and their spleens were removed. Subsequently, the spleen T cells were detected by flow cytometry. Survival curves were generated using the SPSS 24.0 analysis software (SPSS, Inc., Chicago, IL, USA). Institutional Animal Care and Use Committee approval was obtained from the Sun Yat-sen University of Medicine.

### Statistical analysis

SPSS 24.0 and GraphPad Prism version 8.0 (GraphPad Software, San Diego, CA, USA) were used to perform one-way analysis of variance, t-tests (with Welch’s correction for unequal variances), and Kruskal–Wallis test, depending on the data distribution (normal distribution or not). p-values <0.05 were treated as statistically significant (*p < 0.05, **p < 0.01, ***p < 0.001).

## Results

### Characteristics of healthy donors and acute lymphoblastic leukemia patients

PB samples from 15 ALL patients, umbilical cord blood samples from 22 healthy donors, and PB samples from 10 healthy donors were collected. Seven ALL patients were female (46.7%) and eight were male (53.3%), whereas three healthy donors were female (30.0%) and seven were male (70.0%). The median age was 7 (range, 2–14) years in ALL patients and 25 (range, 5–30) in healthy donors. Eight samples were collected from 11 high-risk patients during consolidation therapy, 3 after completion of reinduction chemotherapy, and 4 after receiving the BFM2002 relapse regimen (**Tables 1–1**, **1–2**).

**Table 1-1:** Characteristics of ALL patients and healthy donors.

Variates	ALL patient (n = 15)	Healthy donor (n = 10)
Sex, %		
Male	8 (53.3%)	7 (70.0%)
Female	7 (46.7%)	3 (30.0%)
Age (median, range, years)	7 (2–14)	25 (5–30)

ALL, acute lymphoblastic luekemia.

**Table 1-2:** Chemotherapy administered to ALL patients.

Diagnosis and Treatment	ALL Patients (n = 15)
Diagnosis	
ALL (HR)	11 (73.3%)
R/R ALL	4 (26.7%)
Chemical cycles	
Consolidation	8 (53.3%)
Reinduction	3 (20.0%)
ALL-REZ BFM 2002 protocols	4 (26.7%)

ALL (HR), high risk of acute lymphoblastic luekemia; R/R ALL, relapsed and refractory acute lymphoblastic luekemia; ALL-REZ, Acute Lymphoblastic Leukemia-Relapse Study; BFM, Berlin-Frankfurt-Münster.

### T-lymphocyte subsets of cord blood, peripheral blood, and peripheral blood (patient)

**Figure 1:**
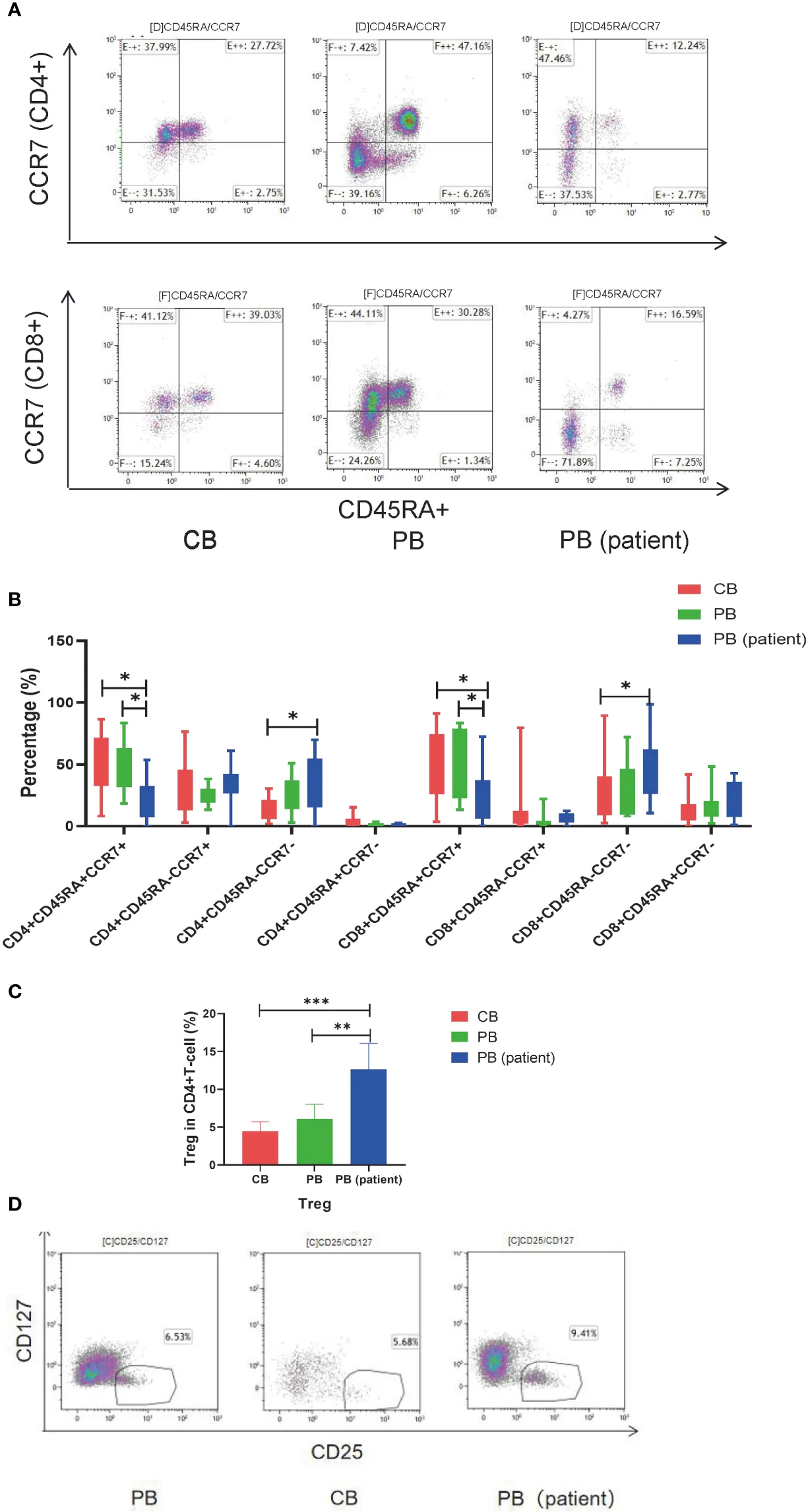
T-cell subsets of CB, PB, and PB (patient) groups. **(A)** Flow cytometry analysis of TN, TCM, TEM, and TEMRA in the three groups of T cells. Right upper quadrant: naive T (CD45RA+CCR7+), left upper quadrant: TCM (CD45RA-CCR7+), left lower quadrant: TEM (CD45RA-CCR7-), and right lower quadrant: TEMRA (CD45RA+CCR7-). **(B)** TN, TCM, TEM, and TEMRA in the three groups of T cells. **(C)** Proportion of Tregs in the three groups of T cells. **(D)** Flow cytometry analysis of Tregs (CD4+CD25+CD127-). TN, Naive T cell; TCM, Central memory T cell; TEM, effector memory T cell; TEMRA, Terminal effector T cell; Treg, regulatory T cell. *p < 0.05, **p < 0.01, ***p < 0.001.

T-cell subsets in the CB, PB, and PB (patient) groups were significantly different. The proportion of CD4+TEM and CD8+TEMRA in the PB (patient) group was significantly higher than those in the other two groups. CD4+TN and CD8+TN were significantly higher in the CB and PB groups than those in the PB (patient) group. The proportion of CD8+TCM was relatively higher in the CB group than those in the PB and PB (patient) groups (**Figures 1A, B**). We also measured the proportion of Treg in the three groups. The proportion of Treg was significantly higher in the PB (patient) group than those in the CB and PB groups (**Figures 1C, D**).

### Antitumor efficacy and cytokine release of cord blood CAR-T, peripheral blood CAR-T, and peripheral blood (patient) CAR-T

**Figure 2:**
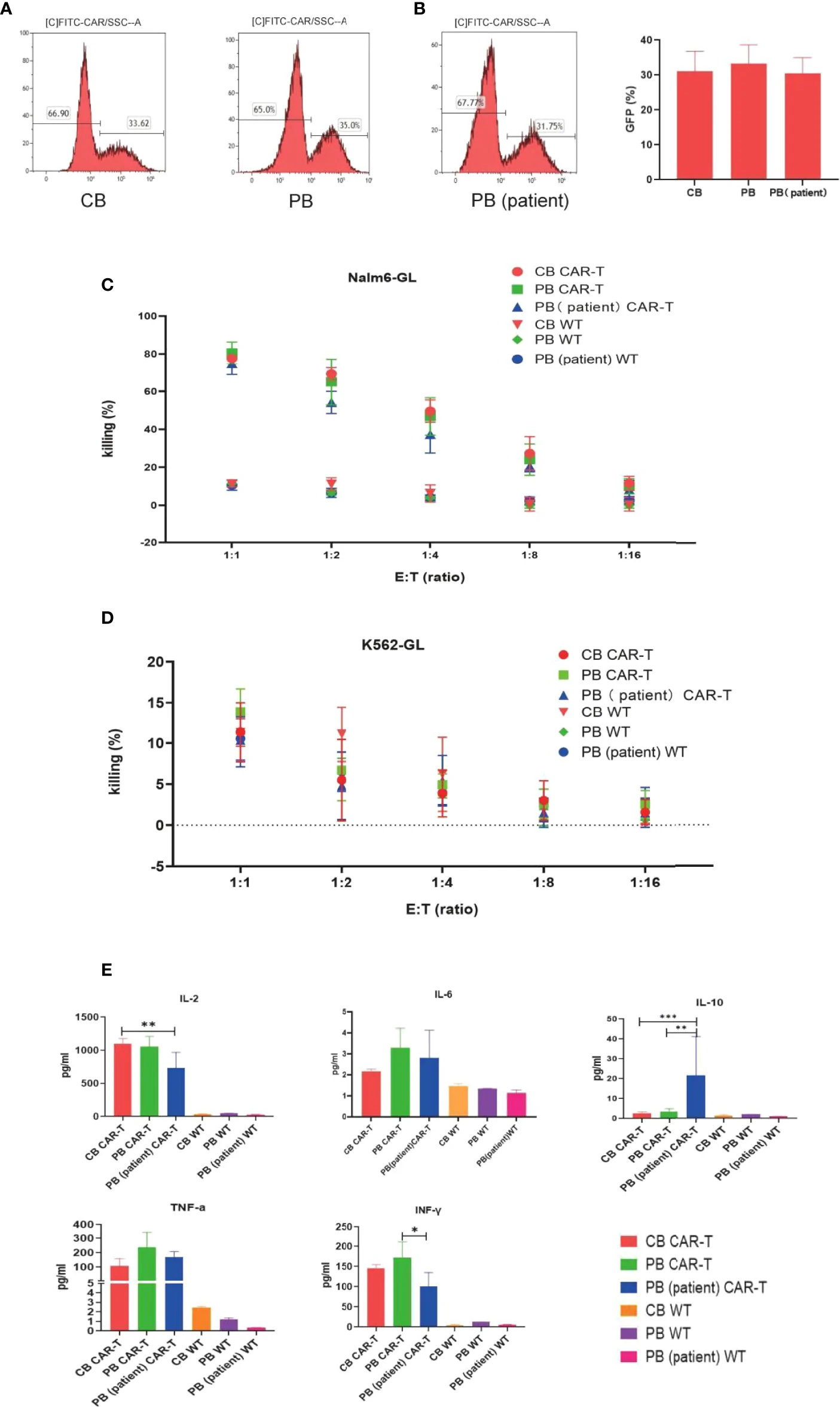
Transfection efficiency and function of CAR-T cells in the three groups. **(A)** CAR expression of T cells in the three groups measured by flow cytometry. **(B)** Transfection efficiency among the three groups. **(C)** Killing efficacy of Nalm6-GL by CB CAR-T, PB CAR-T, PB (patient) CAR-T, and control wild-type T cells after coculture for 18 h Results are representative of at least three independent experiments with T cells from different healthy donors. **(D)** Killing efficacy of K562-GL by CB CAR-T, PB CAR-T, PB (patient) CAR-T, and control wild-type T cells after coculture for 18 h. Results are representative of at least three independent experiments with T cells from different healthy donors. **(E)** Levels of IL-2, IL-6, IL-10, TNF-α, and IFN-γ in the supernatant after the CAR-T cells were cocultured with Nalm6-GL. *p < 0.05, **p < 0.01, ***p < 0.001.

We transfected T cells from 5 different donors of each group into anti-CD19 CAR-T cells. No difference was observed in transfection efficiency among the three groups, which could reach approximately 30% (**Figures 2A, B**).

The anti-CD19 CAR-T cells were collected using flow cytometry, and CB CAR-T, PB CAR-T, and PB (patient) CAR-T cells were cocultured with Nalm6-GL and cocultured with K562-GL as a comparison at effector-to-target ratios of 1:1, 1:2, 1:4, 1:8, and 1:16. The WT T cells of CB, PB, and PB (patient) were treated as the control groups. All three anti-CD19 CAR-T groups had a higher killing efficacy for Nalm6-GL than the control groups, while there were no significance difference in killing efficacy for Nalm6-GL among the CB CAR-T, PB CAR-T, and PB (patient) CAR-T groups. The killing efficacy rates of CB CAR-T, PB CAR-T, and PB (patient) CAR-T were 78.9% ± 1.69%, 80.90% ± 5.2%, and 76.6% ± 1.7%, respectively (p > 0.05). There was no significant difference in the killing efficacy for K562-GL between anti-CD19 CAR-T groups and WT T-cell control groups; the killing efficacy for K562-GL was lower than that for Nalm6-GL (**Figures 2C, D**).

From the concentrations measured in the supernatant, we found that CAR-T cells from all three sources secreted higher levels of IL-2, TNF-α, and IFN-γ than WT T cells after coculture with Nalm6-GL tumor cells. CB CAR-T and PB CAR-T cells secreted higher levels of IL-2 and IFN-γ after coculture with Nalm6-GL tumor cells than PB (patient) CAR-T cells. It is worth noting that IL-10 was detected in the supernatant. PB (patient) CAR-T cells secreted more IL-10 than CB CAR-T or PB CAR-T cells (**Figure 2E**).

### T-cell subsets of cord blood CAR-T, peripheral blood CAR-T, and peripheral blood (patient) CAR-T after coculture with Nalm6-GL

**Figure 3:**
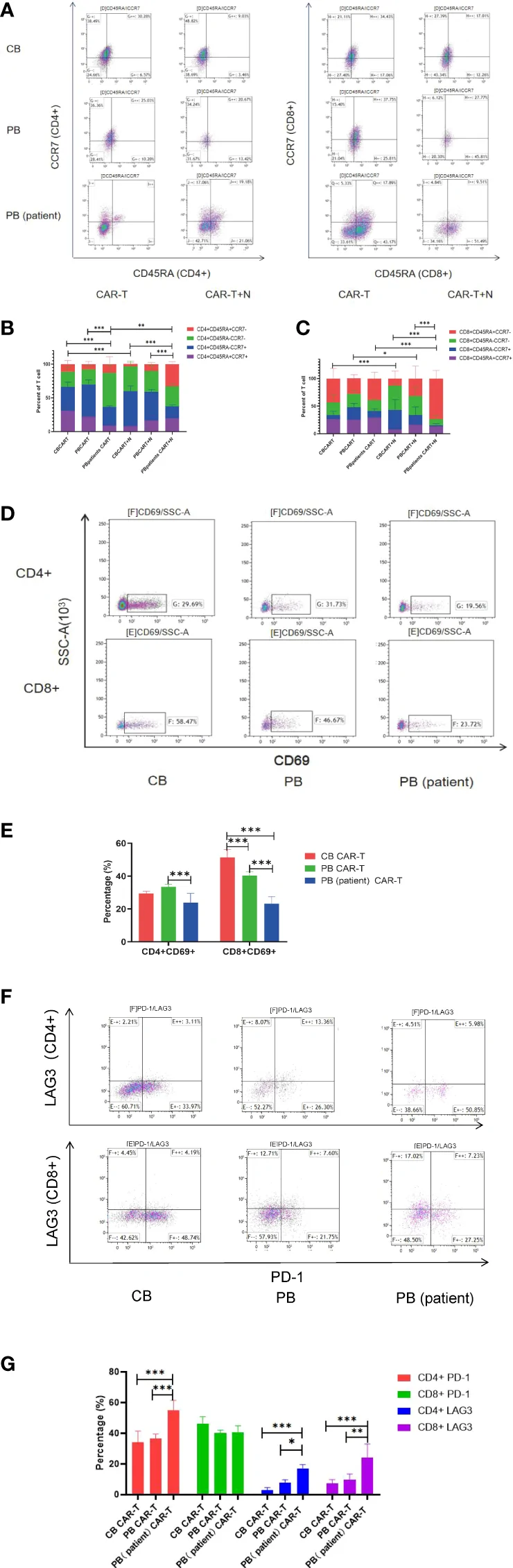
Changes in T-cell subsets, activation, PD-1, and LAG3 expression of CAR-T cells in the three groups after coculture with Nalm6-GL. **(A)** Flow cytometry analysis of TN, TCM, TEM, and TEMRA in CAR-T cells of the three groups. Right upper quadrant: naive T(CD45RA+CCR7+), left upper quadrant: TCM(CD45RA-CCR7+), left lower quadrant: TEM(CD45RA-CCR7-), and right lower quadrant: TEMRA(CD45RA+CCR7-). **(B, C)** Proportion of TN, TCM, TEM, and TEMRA in CAR-T cells. **(B)** CD4+T cells, **(C)** CD8+T cells, red represents TEMRA, green represents TEM, blue represents TCM, and purple represents naive T cells. **(D)** Flow cytometry analysis of CD69 in the three groups of CAR-T cells after coculture with Nalm6-GL for 18 h **(E)** Expression of CD69 in the three groups of CAR-T cells after coculture with Nalm6-GL for 18 h **(F)** Flow cytometry analysis of the expression of PD-1 and LAG3 in the three groups of CAR-T cells after coculture with Nalm6-GL for 72 h **(G)** The expression of PD-1 and LAG3 in the three groups of CAR-T cells after coculture with Nalm6-GL for 72 h. PD-1, programmed death 1; LAG3, Lymphocyte-activation gene 3; TN, Naive T cell; TCM, Central memory T cell; TEM, effector memory T cell; TEMRA, Terminal effector T cell; Treg, regulatory T cell. *p < 0.05, **p < 0.01, ***p < 0.001.

We characterized the T-cell subsets of the three anti-CD19 CAR-T groups before and after coculture with Nalm6*-*GL. CB CAR-T and PB CAR-T had higher proportions of TN and TCM than PB (patient) CAR-T. After exposure to tumor antigens, all three groups of CAR-T cells could rapidly differentiate into effector T cells. However, CB CAR-T and PB CAR-T cells preserved more memory T cells, especially CD8+TCM in CB CAR-T cells. In contrast, TEMRA was higher in PB (patient) CAR-T cells (**Figures 3A–C**). We detected T-cell activation in response to tumor antigen stimulation in all three groups of anti-CD19 CAR-T cells after coculture with Nalm6-GL for 18 h. CD69, a marker of the early activation of T lymphocytes, showed an increase in its level at 2 h after stimulation and gradually decreased after 24 h. The increase in CD69 in CB CAR-T and PB CAR-T was higher than that in PB (patient) CAR-T, especially in CD8+ T cells (**Figures 3D, E**). We also measured the levels of PD-1 and LAG3 in three anti-CD19 CAR-T groups after coculture with Nalm6-GL for 72 h. The expression levels of PD-1 and LAG3 in PB (patient) CAR-T cells were higher than those in CB CAR-T cells and PB CAR-T cells (**Figures 3F, G**).

### Antitumor efficacy of cord blood CAR-T, peripheral blood CAR-T, and peripheral blood (patient) CAR-T *in vivo*

**Figure 4:**
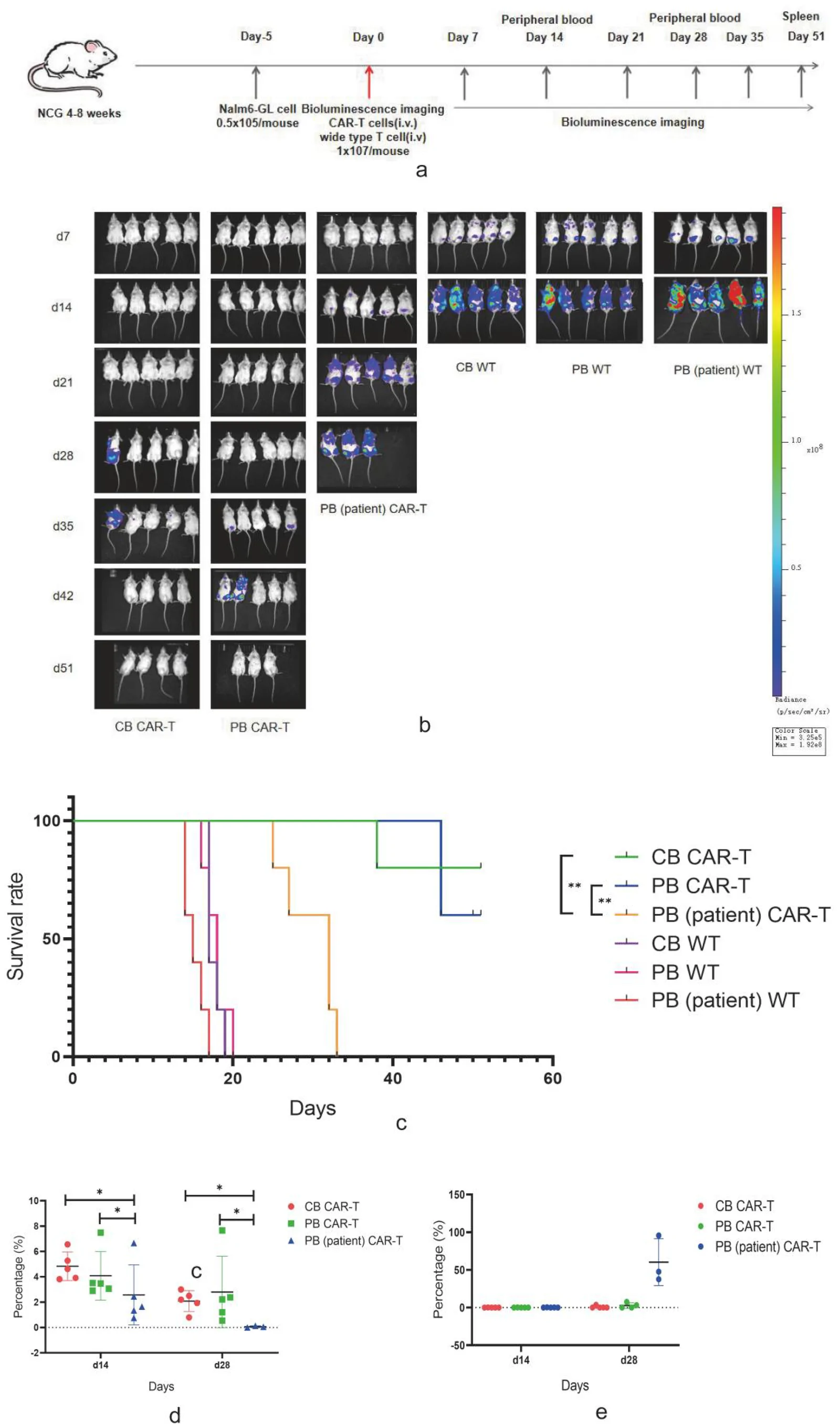
CB CAR-T and PB CAR-T had better antitumor efficacy *in vivo*. **(A)** Flow chart of the *in vivo* experiment. **(B)** Bioluminescence imaging of NALM6-GL-burdened mice treated with the three groups of CAR-T cells and control T cells. **(C)** Survival analysis of mice treated with the three groups of CAR-T cells and control T cells. **(D)** Proportions of CAR-T cells in the peripheral blood of mice on days 14 and 28. **(E)** Proportions of tumor cells in the peripheral blood of mice on days 14 and 28. *p < 0.05, **p < 0.01.

On day 5 after tumor cell injection, the tumor burdens of mice were measured using bioluminescence imaging. Thirty mice were divided into six groups according to tumor load, and these groups were injected with CB CAR-T, PB CAR-T, PB (patient) CAR-T, and WT T cells on day 0 (1 × 10^7^ T cells were injected; CAR-T normalized to 30%). On days 7, 14, 21, 28, 35, 42, and 52, bioluminescence imaging was performed, and on days 14 and 28, the proportion of CAR-T cells and tumor cells in the PB of mice was measured (**Figure 4A**).

We found that the tumor burdens of three WT T cell groups were higher than those of the three CAR-T groups, and the tumor burden of the PB (patient) CAR-T group was higher than those of the CB CAR-T and PB CAR-T groups. At the end of theexperiment, there were four surviving mice in the CB CAR-T group and three in the PB CAR-T group (**Figure 4B**).

The survival analysis showed that the mice of all three CAR-T groups survived longer than the mice in the WT groups, and the mice in the CB CAR-T and PB CAR-T groups survived longer than those in the PB (patient) CAR-T group. The median survival times of the CB CAR-T, PB CAR-T, and PB (patient) CAR-T groups were 51 (39–51) days, 51 (46–51) days, and 32 (25–33) days (p < 0.05), respectively (**Figure 4C**).

We measured the proportion of CAR-T cells and tumor cells in the PB of mice, and the results showed that mice in the CB CAR-T and PB CAR-T groups had a higher proportion of CAR-T cells and a lower proportion of tumor cells in PB than those in mice in the PB (patient) CAR-T groups on days 14 and 28 (**Figures 4D, E**).

### Immunophenotype of three sources of CAR-T cells *in vivo*

**Figure 5:**
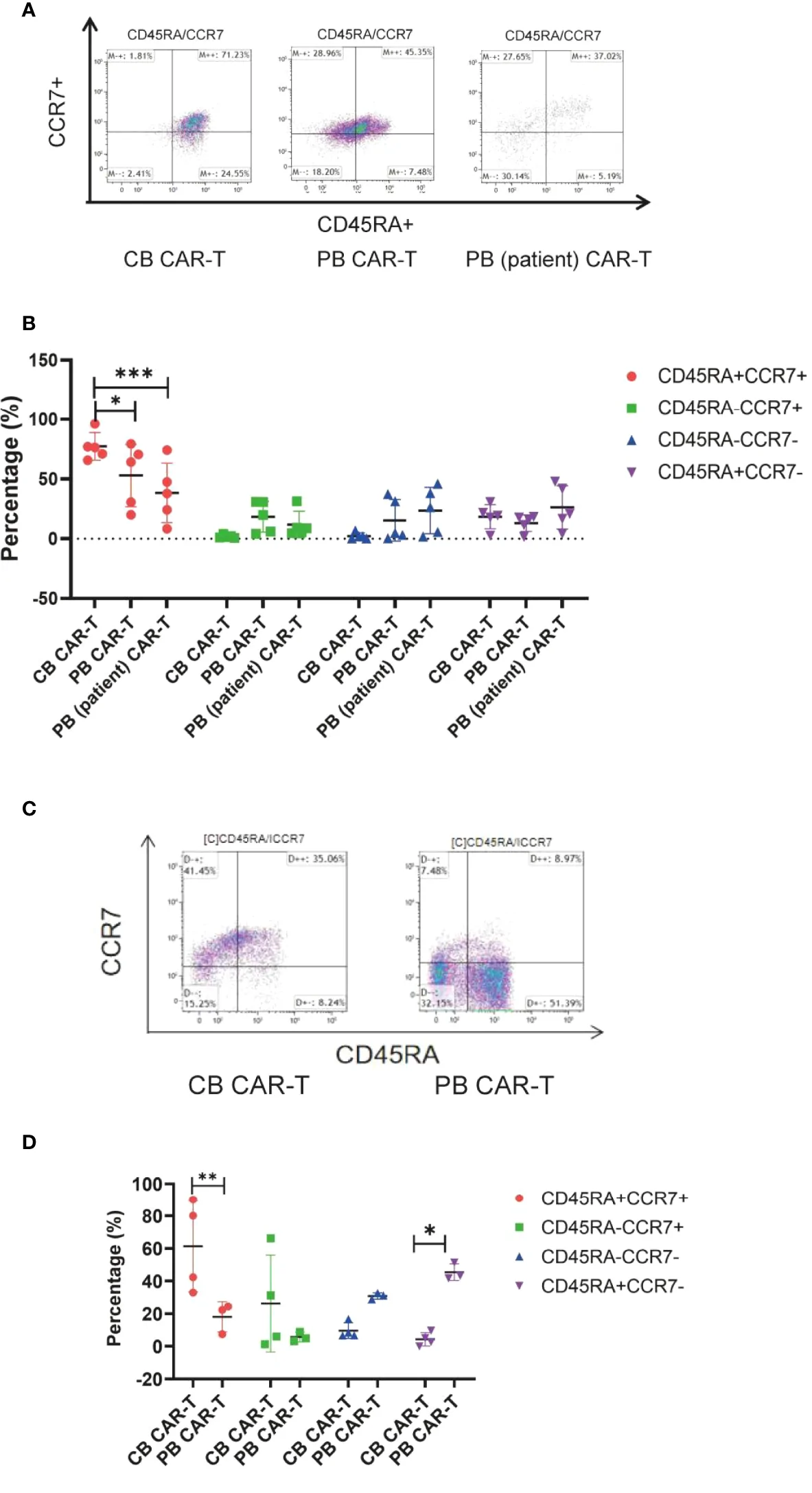
Immunophenotype of the three groups of CAR-T cells *in vivo*. **(A)** Flow cytometry analysis of TN, TCM, TEM, and TEMRA in the peripheral blood of mice in the three CAR-T groups on day 14. Right upper quadrant: naive T(CD45RA+CCR7+), left upper quadrant: TCM(CD45RA-CCR7+), left lower quadrant: TEM(CD45RA-CCR7-), and right lower quadrant: TEMRA(CD45RA+CCR7-). **(B)** Proportion of TN, TCM, TEM, and TEMRA in the peripheral blood of mice in the three CAR-T groups on day 14. Red represents TEMRA, green represents TEM, blue represents TCM, and purple represents naive T cells. **(C)** Flow cytometry analysis of TN, TCM, TEM, and TEMRA in the spleen of mice in the CB and PB CAR-T groups on day 51. **(D)** Proportion of TN, TCM, TEM, and TEMRA in the spleen of mice in the CB CAR-T and PB CAR-T groups on day 51. TN, Naive T cell; TCM, Central memory T cell; TEM, effector memory T cell; TEMRA, Terminal effector T cell; Treg, regulatory T cell. *p < 0.05, **p < 0.01, ***p < 0.001.

We also found that mice in the CB CAR-T and PB CAR-T groups had higher proportions of TN and TCM in their PB on day 14 than mice in the PB (patient) CAR-T group (**Figures 5A, B**). On day 28, the T-cell subsets could not be detected because of a significant decrease in CAR-T cells in the PB. At the end of the experiment, three mice survived in the PB CAR-T group and four in the CB CAR-T group. We characterized the T-cell subsets in the spleen. TN and TCM were the main T-cell subsets in the spleen of mice in the CB CAR-T group, and TEM and TEMRA were the main T-cell subsets in the spleen of mice in the PB CAR-T group (**Figures 5C, D**).

## Discussion

CAR-T cell therapy for R/R B-cell malignancies has achieved good efficacy; however, patients still undergo intensive lymphocytotoxic chemotherapy, leading to imbalance and dysfunction of T-cell subsets, which may affect the antitumor efficacy of CAR-T therapy (15–18). Therefore, the use of allogeneic CAR-T cells to replace autologous CAR-T cells may further improve their antitumor efficacy. Which T-cell subset should be used to prepare allogeneic CAR-T cells remains unclear. CB contains an abundance of immune cells, making it a promising source for cellular immunotherapy. In this study, we compared the immunophenotype and antitumor efficacy of anti-CD19 CAR-T cells derived from CB, PB, and PB (patient) to provide a scientific basis for better use of CAR-T cells derived from CB.

Our results showed that CD4+TN and CD8+TN were significantly higher in the CB and PB groups than those in the PB (patient) group. CD8+TCM was significantly higher in the CB group than those in the PB and PB (patient) groups, whereas the proportions of CD4+TEM and CD8+TEMRA were significantly higher in the PB (patient) group than those in the other two groups. Many studies have investigated the effect of chemotherapy on T cells. Mazur et al. (19) reported that the proportion of CD4+/CD8+T cells, CD45RO+, and CD45RA+ T cells in patients with ALL was imbalanced because of chemotherapy. Other studies have reported that cyclophosphamide and cytarabine selectively damage early-lineage T cells and could also damage the function of the thymus, leading to a long-term decrease in the production of naive T cells (20–22). In addition to malignant hematological diseases, patients with solid tumors also have significant abnormalities in T-cell subsets and functions. Recent studies have reported that patients with solid tumors and lymphomas showed chemotherapy-related depletion of early-lineage T cells, and many pediatric patients with solid tumors had lower numbers of naive T cells and an obvious imbalance of T-cell subsets than B-ALL pediatric patients, reducing the expansion potential associated with successful adoptive cellular therapies (21).

The immunophenotype and functional characteristics of CAR-T cells are associated with the efficacy of CAR-T cell treatment. Effector CAR-T cells could respond rapidly to tumor antigens, whereas naive and memory T cells are key to inhibiting tumor recurrence (23). Our study also showed that CB CAR-T and PB CAR-T cells could preserve a higher proportion of TCM than PB (patient) CAR-T cells following exposure to tumor antigens, which was related to better antitumor efficiency and longer tumor-suppressive effects than those of PB (patient) CAR-T cells in tumor-bearing mice. CB CAR-T cells especially preserved a higher proportion of TN and TCM in the spleen of mice at the end of the *in vivo* experiment. Gattinoni et al. (24) reported that stem cell-like memory T cells (TSCM, CD45RA+CD62L+CD95+) can self-renew and generate differentiated progeny, which could overcome the limitations of current adoptive T-cell therapies, including inefficient T-cell engraftment, persistence, and ability to mediate a prolonged immune attack. Another study has shown that CD8(+)CD45RA(+)CCR7(+) CAR-T cells produce greater antitumor activity mediated by their self-renewal ability, even with repeated antigen stimulation while preserving their migration to secondary lymphoid organs (25).

We also found that the proportion of Tregs in the PB (patient) group was higher than those in the CB and PB groups. We also detected the level of IL-10 in the supernatant following CAR-T cells cocultured with tumor cells and found that the level of IL-10 was higher in the PB (patient) CAR-T group than those in the CB and PB CAR-T groups, which further demonstrates that autologous CAR-T cells in R/R ALL patients have higher Treg levels and could secrete IL-10, which inhibited antitumor effects. Recent studies have revealed that despite strong antigen-specific activation, CD4⁺CD25+ CAR-T cells produce only a weak target cell lysis and lower levels of perforin and granzyme B upon CAR activation, whereas CD4⁺CD25^-^ CAR-T cells are potent killers with higher levels of perforin and granzyme B upon CAR activation (26). Lee et al. (27) reported that CAR-Treg cells efficiently inhibit the proliferation of activated CAR-T cells to lyse CD19(+) Raji tumor cells both *in vivo* and *in vitro*.

In the clinical treatment process, CAR-T cells are constantly exposed to tumor antigens, which leads to the deterioration of CAR-T cell function. Exhausted CAR-T cells have lower proliferation and cytokine production in response to antigen stimulation and, at the same time, express higher inhibitory receptors such as PD-1, TIM3, and LAG3 (28). Gargett et al. (29) reported that the relationship between PD-1 expression and CAR-T cell apoptosis was observed after repeated antigen stimulation, and PD-1 blockade enhanced CAR-T cell survival. In our study, we measured the levels of PD-1 and LAG3 in the three sources of CAR-T cells after coculture with Nalm6-GL. The levels of PD-1 and LAG3 in all three groups of CAR-T cells increased in response to antigen stimulation, especially in PB (patient) CAR-T cells following coculture with Nalm6-GL. This is another indication that autologous CAR-T cells from patients with cancer are more likely to be depleted after exposure to tumor antigens than CAR-T cells from healthy donors.

In addition to the abnormalities of T-cell subsets and function, the use of autologous T cells has other limitations, such as duration of the process sometimes incompatible with uncontrolled hemopathy and extremely high cost. With the development of gene-editing technology, CAR-T cell products “off-the-shelf” will become a reality. CB contains many cells with a higher proportion of naive T cells and negligible damage to the donor, thus making it a promising source for “off-the-shelf” allogeneic CAR-T cells both for patients with hematologic malignancies or solid tumors. Although healthy donor-derived allogeneic CAR-T cell therapy achieved better antitumor efficacy in immunodeficient mice, there are still a number of issues in clinical application (30). First, it may cause severe graft-versus-host disease. Second, the host’s rejection of allogeneic CAR-T cells will also lead to a short retention time of CAR-T cells *in vivo* and a reduced therapeutic effect. For this reason, allogeneic hematopoietic stem cell transplantation with donor-derived allogeneic CAR-T cells may also be a good choice for patients with R/R B-ALL, and many clinical studies achieved good results without significant CAR-T cell therapy-related side effects (31, 32).

In conclusion, patients with malignant tumors will have abnormalities of T-cell subsets and function. CAR-T cells derived from CB had a higher proportion of naive T and TCM, better antitumor efficacy, and longer tumor-suppressive effect in a leukemia mouse model than those of CAR-T cells derived from R/R ALL patients, which make it a promising source for allogeneic CAR-T cells.

## Data availability statement

The raw data supporting the conclusions of this article will be made available by the authors, without undue reservation.

## Ethics statement

The studies involving human participants were reviewed and approved by the Institutional Review Board of the Sun Yat-sen Memorial Hospital, Clinical Research Center. Written informed consent to participate in this study was provided by the participants’ legal guardian/next of kin. The animal study was reviewed and approved by the Committee on Animal Use and Care of Sun Yat-sen University.

## Author contributions

A complete list of participating institutions has been provided in the Appendix. J-PF and L-HX conceived the idea of the study. D-DL, YL, and W-CH performed the experiments. D-DL analyzed the data and wrote the first manuscript draft. H-C, X-YL, W-XL, L-WZ, K-YQ, and H-QY made suggestions and polished the language. J-PF and L-HX reviewed the manuscript, and J-PF provided financial help. All the authors have read and approved the final manuscript.

## Funding

This study was supported by the Basic and Applied Basic Research Foundation of Guangdong province (2021A1515010240).

## Conflict of interest

The authors declare that the research was conducted in the absence of any commercial or financial relationships that could be construed as a potential conflict of interest.

## Correction note

A correction has been made to this article. Details can be found at: 10.3389/fonc.2026.1982369.

## Publisher’s note

All claims expressed in this article are solely those of the authors and do not necessarily represent those of their affiliated organizations, or those of the publisher, the editors and the reviewers. Any product that may be evaluated in this article, or claim that may be made by its manufacturer, is not guaranteed or endorsed by the publisher.
