# Correction: Umbilical cord blood: a promising source for allogeneic CAR-T cells

**DOI:** 10.3389/fonc.2026.1982369

**Published:** 2026-09-18

**Authors:** Dian-Dian Liu, Wei-Cong Hong, Kun-Yin Qiu, Xin-Yu Li, Yong Liu, Li-Wen Zhu, Wei-Xin Lai, Han- Chen, Hua-Qing Yang, Lu-Hong Xu, Jian-Pei Fang

**Affiliations:** 1 Department of Pediatrics, Sun Yat-sen Memorial Hospital, Sun Yat-sen University, Guangzhou, China; 2 Key Laboratory of Malignant Tumor Gene Regulation and Target Therapy of Guangdong Higher Education Institutes, Sun Yat-sen University, Guangzhou, China

**Keywords:** chimeric antigen receptor T cells, umbilical cord blood, peripheral blood, acute lymphoblastic leukemia, T-lymphocyte subsets, NCG-immunodeficient mice

There was a mistake in **Figure 4B** as published the panel corresponding to the CB CAR-T group at day 21 was inadvertently misused. The corrected **Figure 4** appears below.

**Figure 4:**
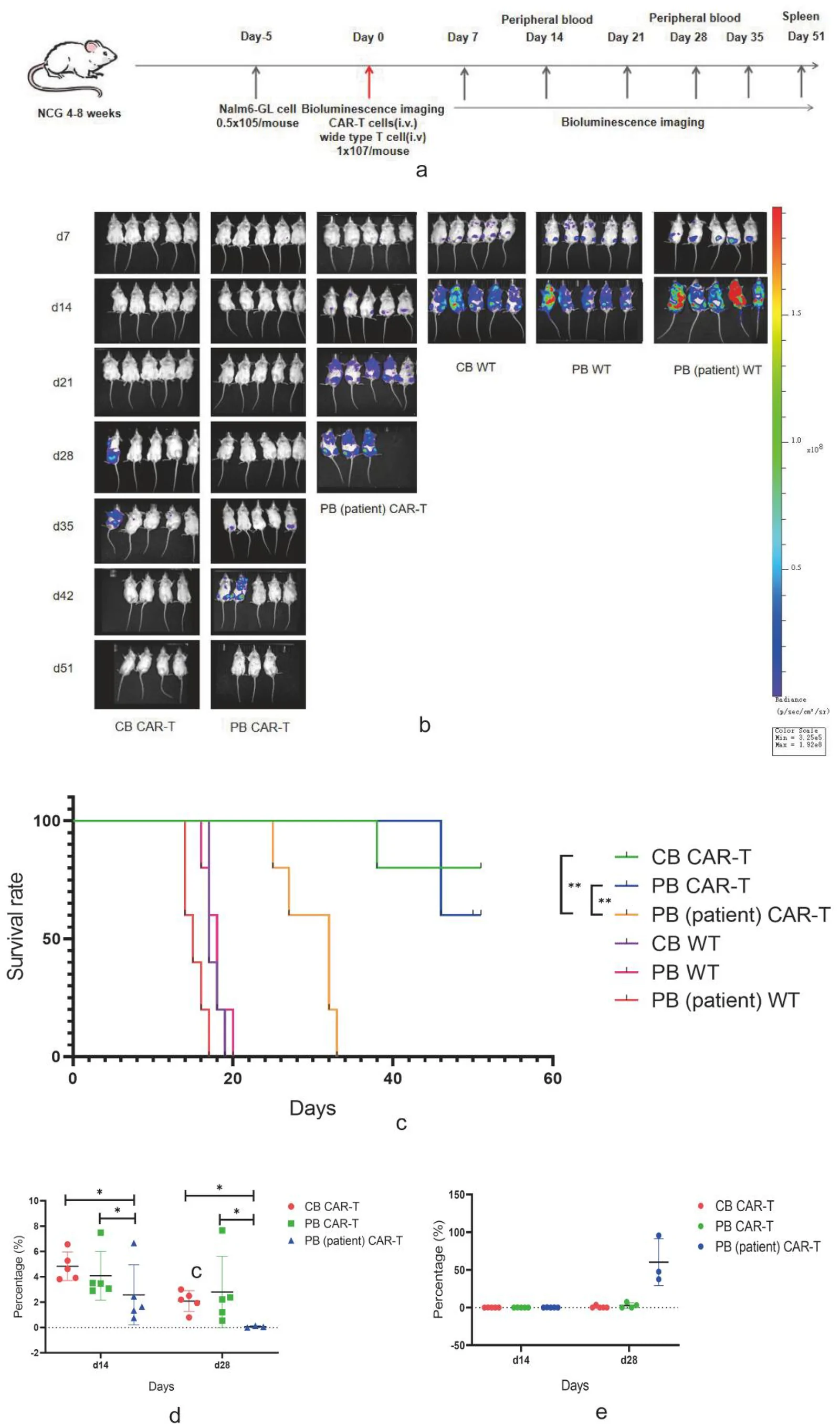
CB CAR-T and PB CAR-T had better antitumor efficacy in vivo. **(A)** Flow chart of the in vivo experiment. **(B)** Bioluminescence imaging of NALM6-GL-burdened mice treated with the three groups of CAR-T cells and control T cells. **(C)** Survival analysis of mice treated with the three groups of CAR-T cells and control T cells. **(D)** Proportions of CAR-T cells in the peripheral blood of mice on days 14 and 28. **(E)** Proportions of tumor cells in the peripheral blood of mice on days 14 and 28. *p < 0.05, **p < 0.01.

In the Statistical analysis section, “Kruskal–Wallis Friedman tests” has been corrected to “Kruskal–Wallis test,” “one-way analysis of variance” has been corrected to “one-way ANOVA,” and “signifificant” has been corrected to “significant.”

The correction has been made to the section **Materials and methods**, *Statistical analysis*:

“SPSS 24.0 and GraphPad Prism version 8.0 (GraphPad Software, San Diego, CA, USA) were used to perform one-way analysis of variance, t-tests (with Welch’s correction for unequal variances), and Kruskal–Wallis test, depending on the data distribution (normal distribution or not). p-values <0.05 were treated as statistically significant (*p < 0.05, **p < 0.01, ***p < 0.001).”

In the **Results** section, *Antitumor efficacy of cord blood CAR-T, peripheral blood CAR-T, and peripheral blood (patient) CAR-T in vivo*, Paragraph 2, line 4 to 6. the numbers of surviving mice in the CB CAR-T and PB CAR-T groups were reversed and have been corrected.

“At the end of the experiment, there were three surviving mice in the CB CAR-T group and four in the PB CAR-T group (**Figure 4B**).” has been corrected to”At the end of the experiment, there were four surviving mice in the CB CAR-T group and three in the PB CAR-T group (**Figure 4B**).”

The original version of this article has been updated.

